# Genome Sequences of Human and Livestock Isolates of *Brucella melitensis* and *Brucella abortus* from the Country of Georgia

**DOI:** 10.1128/genomeA.01518-16

**Published:** 2017-02-09

**Authors:** Ketevan Sidamonidze, Jun Hang, Yu Yang, George Dzavashvili, Ekaterine Zhgenti, Nino Trapaidze, Paata Imnadze, Mikeljon P. Nikolich

**Affiliations:** a National Center for Disease Control & Public Health, Tbilisi, Georgia; b Ivane Javakhishvili Tbilisi State University, Tbilisi, Georgia; c Walter Reed Army Institute of Research, Silver Spring, Maryland, USA; d U.S. Army Medical Research Unit, Georgia, Tbilisi, Georgia

## Abstract

Brucellosis, which is among the most widespread global zoonotic diseases, is endemic in the nation of Georgia and causes substantial human morbidity and economic loss. Here, we report whole-genome sequences of three *Brucella melitensis* and seven *Brucella abortus* isolates from cattle, sheep, and humans that represent genetic groups discovered in Georgia.

## GENOME ANNOUNCEMENT

Brucellosis is one of the most globally common zoonotic diseases, with more than 500,000 human cases reported worldwide annually (1). Brucellosis epidemiology changes under various sanitary, socioeconomic, and political conditions. The genus *Brucella* comprises facultative intracellular bacterial pathogens that can infect a wide range of mammals, including humans, livestock, rodents, and marine mammals (2, 3). Five *Brucella* species are known to be pathogenic for humans: *B. melitensis*, *B. abortus*, *B. suis*, *B. canis*, and *B. maris* (4). Among these, *B. abortus* and *B. melitensis* are classified as category B biological threat agents (5) (https://emergency.cdc.gov/agent/agentlist.asp).

Molecular typing assays are routinely used to genetically characterize *Brucella* isolates and determine clonal associations, and thus provide a means to trace-back to sources of infection, and can also be used to discriminate naturally occurring outbreaks from a bioterrorism event. The genetic typing tool multiple-locus variable-number tandem-repeat analysis (MLVA) can provide high-resolution genetic subtyping information for accurate epidemiological investigations (6). In this study, we used a 15-marker MLVA system (7) to subtype *Brucella* strains isolated in Georgia between 2010 and 2013. Based on this analysis, 10 isolates, including three *B. melitensis* and seven *B. abortus* strains, were selected to represent major genetic clusters for whole-genome pyrosequencing (Table 1). Purified *Brucella* genomic DNA samples were sheared to around 1-kb-long fragments using the Covaris S2 system (Covaris, Woburn, MA). The shotgun library of DNA fragments for each sample was prepared and sequenced using Roche GS FLX sequencing system and reagents (Roche 454 Life Sciences, Branford, CT). Sequence read data were successfully assembled into *de novo* assembly contigs using Roche GS Assembler software (Newbler), with most sequence reads assembled and high sequence alignment depths achieved (Table 1). The size of each draft genome, as estimated based on the length and copy number of every contig, is close to the expected length of 3.3 Mb. The sequences share high nucleotide identity (>99%) with respective known *Brucella* genome sequences, including GenBank reference genomes (RefSeq) *B. abortus* S19 (accession numbers NC_010740 and NC_010742) and *B. melitensis* M28 (accession numbers NC_017244 and NC_017245). The draft genomes were annotated by utilizing the NCBI Prokaryotic Genome Annotation Pipeline (PGAP, revision 33 [http://www.ncbi.nlm.nih.gov/genomes/static/Pipeline.html]) (Table 1).

**TABLE 1:** *Brucella* genomes and annotations

Strain	Collection date (yr)	Source of isolation	GenBank accession no.	No. of *de novo* contigs	Fold coverage depth	Contig *N*_50_ (bp)	No. of CDSs^a^
*B. abortus* 1247/10-Geo	2010	Bovine blood	MIJH00000000	28	36.4	364,279	3,035
*B. abortus* 1549/11-Geo	2011	Bovine milk	MIJI00000000	30	26.8	254,267	3,032
*B. abortus* 1844/12/12-Geo	2012	Human blood	MIJJ00000000	26	38.0	390,977	3,032
*B. abortus* 1910/13-2013-Geo	2013	Human blood	MIJK00000000	30	34.0	364,276	3,030
*B. abortus* 1238-10-Geo	2010	Ovine blood	MIJL00000000	29	47.3	251,385	3,039
*B. abortus* 1236-10-Geo	2010	Bovine milk	MIJM00000000	26	52.9	364,304	3,031
*B. abortus* 375-10-Geo	2010	Human blood	MIJN00000000	30	76.3	391,124	3,038
*B. melitensis* 1771/12-Geo	2012	Human blood	MIJO00000000	32	53.7	222,046	3,012
*B. melitensis* 1252/10-Geo	2010	Bovine milk	MIJP00000000	29	54.4	250,822	3,010
*B. melitensis* 1268/11-Geo	2011	Bovine milk	MIJQ00000000	32	69.9	298,955	3,018

a CDSs, protein-coding sequences.

Brucellosis remains a major agricultural and public health problem in the nation of Georgia (8, 9). Acquisition of genome sequences for representative genetic variants of the two most important pathogenic *Brucella* species will enable genome-wide phylogenetic and polymorphism analyses to enhance brucellosis surveillance in Georgia. To our knowledge, these are the first published whole-genome sequences of *Brucella* isolates from Georgia or the broader South Caucasus region. Work under way includes comparative analyses of these and other *Brucella* genomes to identify unique single nucleotide polymorphisms (SNPs) and genome structural variations for understanding of *Brucella* pathogenicity and the application of this genomic information to brucellosis epidemiology and disease control.

### Accession number(s).

The whole-genome sequences for *B. abortus* and *B. melitensis* were deposited in GenBank under BioProject numbers PRJNA338234 and PRJNA339926, respectively, with accession numbers listed in Table 1.
